# A new species of Chelonus (Areselonus) (Hymenoptera, Braconidae) from India reared from *Acrocercops lysibathra* (Meyrick) on *Cordia latifolia* Roxb.

**DOI:** 10.3897/zookeys.737.20835

**Published:** 2018-02-12

**Authors:** Zubair Ahmad, Hamed A. Ghramh

**Affiliations:** 1 Department of Biology, College of Sciences, King Khalid University, PO Box-9004, Abha-61413. Kingdom of Saudi Arabia; 2 Research Centre for Advance materials Science (RCAMS), King Khalid University, PO Box-9004, Abha-61413. Kingdom of Saudi Arabia

**Keywords:** *Areselonus*, Braconidae, *Chelonus*, *Cordia latifolia*, Hymenoptera, India, Parasitoids, leaf miners *Acrocercops lysibathra*

## Abstract

Chelonus (Areselonus) spinigaster **sp. n.**, (Hymenoptera: Braconidae: Cheloninae) is described from India. The new species was reared from the moth species *Acrocercops lysibathra* (Meyr.) on *Cordia latifolia* Roxb.

## Introduction

Leaf mining insects are considered as serious pests if leaves of crops are extensively attacked, harvests are reduced or seedling plants even totally destroyed (Spencer 1990). The majority of the leaf mining larvae belong to the Lepidoptera or Diptera, followed to a lesser degree by Coleoptera and Hymenoptera (Csoka 2003). Among the lepidopteran leaf miners, the family Gracillariidae includes several species which are considered serious pests in several parts of the world (Davis 1987). Parasitoid insects play the most important role as natural enemies of leaf miners. In some cases, they cause more than 90 % mortality and have great potential in biological pest control programs (Hawkins et al. 1993). Parasitoids of leaf miners belong exclusively to the order Hymenoptera. Among the parasitoid Hymenoptera chalcidoid wasps (superfamily Chalcidoidea), Ichneumonidae and Braconidae (superfamily Ichneumonoidea) are the most important groups associated with leaf miners.

As far as India is concerned, there is a lack of information on braconid parasitoids especially reared from leaf mining lepidopteran hosts. Wilkinson (1928) described two microgastrine parasitoids, viz., *Apanteles bambusae* Wilkinson and *Apanteles calycinae* Wilkinson of the leaf miner *Cosmopteryx bambusae* (Meyrick) and *Acrocercops supplex* (Meyrick). Subsequently, Nixon (1940) added two hormiine parasitoids to the list of lepidopteran leaf miners, viz., *Parahormius jason* and *Paraharmius deiphobus*. Chaterjee and Misra (1974) added *Epicephalacha lybachma* (Meyr.) to the host list of *Bracon lefroyi* (Dudgeon and Goughs). Recently, Walker and Huddleston (1987) and Shujauddin and Varshney (1997) described chelonine parasitoids associated with lepidopteran leaf miners in India.

The subgenus Areselonus of the genus *Chelonus* Panzer (Braconidae: Cheloninae) was named by Braet (1999) to accommodate species in which the carapace ends in an apical spine-like protuberance and vein SR1 of the fore wing is reduced. This subgenus contains only three described species (Yu et al. 2012). In the present article a new species is described from India and a key to the species of subgenus Areselonus is given.

## Materials and methods

This study was conducted in the vicinity of western Uttar Pradesh (North India) in order to identify parasitoids of leaf miners along the roadside at AMU campus. The parasitoids were reared in the laboratory in glass rearing jars measuring 8”× 4” at 25 °C +2 with 70 % R.H. in the insectory. The leaves with lepidopteran leaf miners were collected from the plants and transferred to the jars. A complete data set including the date of collection, locality, and name of host plant was maintained. The emerged parasitoids were preserved initially in 75 % alcohol with a few drops of glycerol. These specimens were later mounted on cards. The reared parasitoids were separated based on morphological characters. One braconid species was studied in this research. We followed van Achterberg (1993) for the terminologies of various body parts and wing venation and Eady (1968) for terminology of surface sculpture. The specimens were deposited in the Insect Collection of the Department of Zoology, Aligarh Muslim University, Aligarh, India (ZDAMU).

## Results and discussion

### Key to species of the subgenus Areselonus Braet (modified after Braet 1999)

**Table:** 

1	Apical spine of metasoma large, only basally setose and with pores apically	**2**
–	Apical spines small, entirely setose and without pores apically	**3**
2	Mesopleuron densely and finely punctate posteriorly; ovipositor sheath less than 0.1 times as long as fore wing	**C. (A.) minutissimus Braet, 1999**
–	Mesopleuron smooth and sparsely punctate posteriorly; ovipositor sheath more than 0.1 times as long as fore wing	**C. (A.) missai Braet, 1999**
3	Metasoma strongly declivous below apical spine; clypeus rugose	**C. (A.) spinigaster sp. n.**
–	Metasoma angled with apical spine, hardly or not declivous below spine; clypeus sparsely punctate	**C. (A.) chailini Walker & Huddleston, 1987**

#### Chelonus spinigaster sp. n.

Taxon classificationAnimaliaHymenopteraBraconidae

http://zoobank.org/E24FBAA6-A4BE-4E6B-9CB7-94AD497FB692

Figs 1 , 2–8

##### Material examined.

Holotype, ♀, INDIA: Uttar Pradesh, Aligarh, 11. VIII. 1980., ex. *Acrocercop lysibathra* on *Cordia latifolia* Roxb. (coll. Shujauddin) (HB-364, ZDAMU). Paratypes, 7♀, 14 ♂♂, with same data as holotype (HB-364, ZDAMU).

**Figure 1.**
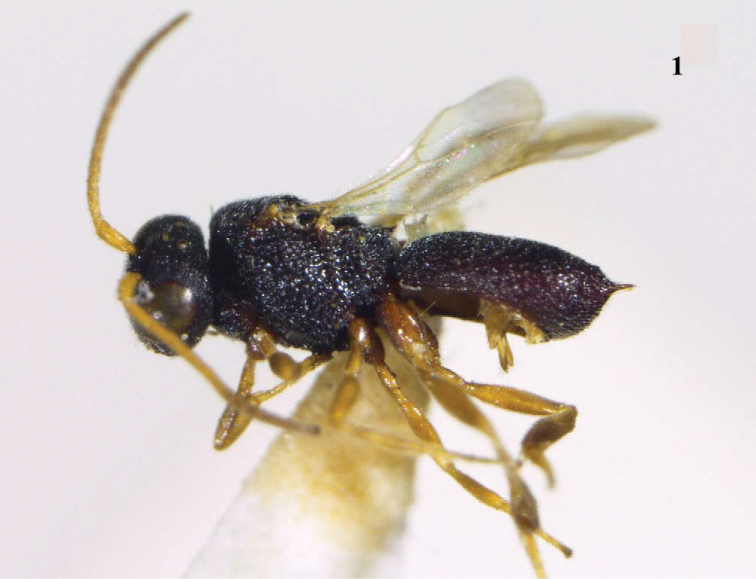
*Chelonus spinigaster* sp. n., female, holotype, habitus, lateral aspect.

##### Diagnosis.

Chelonus (Areselonus) spinigaster sp. n. is closely related to *C. (A.) chailini* (Walker & Huddleston, 1987) but differs from it in having the metasoma strongly declivous below the spine (metasoma angled with apical spine, hardly or not declivous below spine in *C. (A.) chailini*); clypeus rugose (clypeus sparsely punctate in *C. (A.) chailini*); and the wings hyaline (wings partially infuscate apically in *C. (A.) chailini*).

##### Description.

Holotype. Female: Body length: 2.4 mm.

***Head*** 1.6× as wide as long; eye 1.7× as long as temple in dorsal view; frons strigose, slightly depressed, carina distinct; OOL = 1.5× POL; face rugulose, 1.8× as wide as high, carina absent; clypeus rugose; malar space twice basal width of mandible, latter with two subequal teeth; antenna 16-segmented, subfiliform, extending back slightly beyond base of metasoma, scape twice as long as broad, F1 almost 3.0× as long as wide, this ratio decreases gradually, F8–F11 almost as long as wide, F12–F13 slightly longer than wide and apical segment twice as long as wide.

***Mesosoma*** 1.2× as long as wide in lateral view; mesoscutum reticulate-rugose, notauli shallow; scutellum reticulate; propodeum reticulate-rugose, lateral pair of tubercles almost as long as submedian pair.

***Wings***: Fore wing shorter than body; pterostigma twice as long as wide, slightly longer than 1-R1; 3-SR 1.6× as long as r; SR1 curved.

***Legs***: Hind femur 3.3× as long as broad, 0.8× as long as hind tibia, hind tibia 1.3× as long wide and 1.3× longer than hind tarsus.

***Metasoma*** 2.8× as long as high in lateral view, posteriorly distinctly less than twice as high as basally, strongly convex medially, reticulate-rugose with converging carinae on basal fourth and a small spine at apex; ventral opening not reaching apex, distance from ventral opening to apex of metasoma 1.7× as long as hind basitarsus; ovipositor sheaths in lateral view almost as long as hind basitarsus; metasoma strongly declivous below apical spine (Figs 7, 8).

**Figures 2–8.**
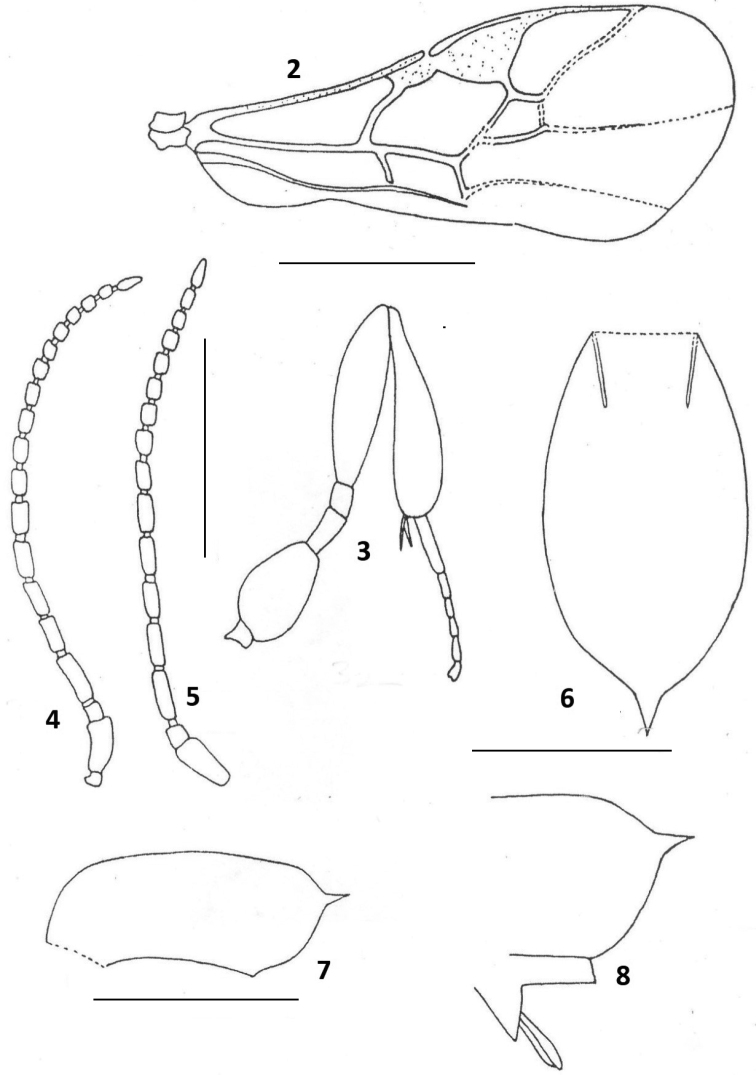
*Chelonus spinigaster* sp. n., female, holotype. **2** Fore wing **3** hind leg **4** antenna of female **5** antenna (of male) **6** metasoma, dorsal aspect **7** metasoma, lateral view **8** apex of metasoma, lateral aspect.

***Colour***: Head and mesosoma black; antenna yellow, gradually becoming brown towards apex; eyes black with yellowish tint; ocelli brownish black, stemmaticum black; metasoma brownish black; apical spine of metasoma and legs brown with fore and mid tibiae and tarsi yellowish, coxae blackish brown; wings hyaline, pterostigma, parastigma, veins C+SC+ R and 1-R1 brown, rest of veins slightly pigmented.

Male: Similar to female except rather elongated antenna; apex of metasoma devoid of a foramen.

##### Host.

*Acrocercops lysibathra* (Meyrick).

##### Distribution.

India: Uttar Pradesh.

##### Etymology.

The species name refers to the presence of a spine on the metasoma.

## Supplementary Material

XML Treatment for Chelonus spinigaster
